# Consumption

**Published:** 1880-12

**Authors:** 


### CONSUMPTION.

Consumption usually begins with a slight, dry cough in the morning, then, on going to bed, getting more and more frequent, with more and more phlegm, increasing debility, thinness of flesh, shortness of breath and quickening pulse. In fatal cases, its average course is about two years ; hence the importance of arresting the disease at as early a stage as possible, and the sooner rational means are employed for this purpose, the greater the chances of success.

The disease is owing to an irritation commencing in the throat and extending to the lungs, so that their action is interfered with, and the blood does not receive sufficient oxygen to purify it. The first thing to be done is to remove the obstruction, which is the irritation or congestion of the lungs. Four ounces of glycerine, two ounces of alcohol, two ounces of water and one grain of morphine make an excellent mixture for relieving the cough. It should be taken in doses of two teaspoonfuls every two hours until the cough is relieved.

The chest, just below the neck, should be rubbed with tartar-emetic ointment every morning over a space as large as the hand, until a thick crop of sores is brought out; then rub the ointment between the sores to bring out a new crop. Meantime the patient should take regular and vigorous exercise in the open air. There is nothing that equals horseback riding as a remedy for this disease. If a consumptive were to "live in the saddle" and sleep out of doors, taking care to keep the feet dry and warm, and to live upon good, nourishing food; in short, to "rough it," he would recover his health in a few months, even if the disease had made considerable progress. The trouble is that it requires a strong will to carry out so severe a course, in spite of the languor and debility which disposes an invalid to quiet despondency.

The most marked sign of lung disease is emaciation; and the most positive indication of returning health is increase in weight.

---

We have just read a handkerchief flirtation code, and advise all men desiring to avoid breach of promise suits to wipe their mouths with their coat-tails.—*Philadelphia Telegraph.*

---

The *Gaulois* announces that "the prescriptions of supreme *chic* formally prohibit giving an arm to a lady under any other circumstances than on entering the dining-room."

